# Spin-Labeled Diclofenac: Synthesis and Interaction with Lipid Membranes

**DOI:** 10.3390/molecules28165991

**Published:** 2023-08-10

**Authors:** Denis S. Baranov, Anna S. Kashnik, Anastasiya N. Atnyukova, Sergei A. Dzuba

**Affiliations:** 1 Voevodsky Institute of Chemical Kinetics and Combustion, Russian Academy of Sciences, 630090 Novosibirsk, Russia; baranov@kinetics.nsc.ru (D.S.B.); anna.smor.mr@gmail.com (A.S.K.); 2 Department of Physics, Novosibirsk State University, 630090 Novosibirsk, Russia; a.atnyukova@g.nsu.ru

**Keywords:** non-steroidal anti-inflammatory drug, NSAID, lipid bilayer, EPR, ^2^H ESEEM

## Abstract

Diclofenac is a non-steroidal anti-inflammatory drug (NSAID) from the group of phenylacetic acid derivatives, which has analgesic, anti-inflammatory and antipyretic properties. The interaction of non-steroidal anti-inflammatory drugs with cell membranes can affect their physicochemical properties, which, in turn, can cause a number of side effects in the use of these drugs. Electron paramagnetic resonance (EPR) spectroscopy could be used to study the interaction of diclofenac with a membrane, if its spin-labeled analogs existed. This paper describes the synthesis of spin-labeled diclofenac (diclofenac-SL), which consists of a simple sequence of transformations such as iodination, esterification, Sonogashira cross-coupling, oxidation and saponification. EPR spectra showed that diclofenac-SL binds to a lipid membrane composed of palmitoyl-2-oleoyl-sn-glycero-3-phosphocholine (POPC). ^2^H electron spin echo spectroscopy (ESEEM) was used to determine the position of the diclofenac-SL relative to the membrane surface. It was established that its average depth of immersion corresponds to the 5th position of the carbon atom in the lipid chain.

## 1. Introduction

Non-steroidal anti-inflammatory drugs are effective antipyretic and analgesic pharmacological agents [1], which are also used in the treatment of other diseases, including cancer [2], arthritis [3,4] and neurodegenerative diseases [5]. The most commonly used NSAIDs are ibuprofen, naproxen and diclofenac [6]. Due to their wide application, these compounds are found even in the environment in the form of human waste [7].

Like all NSAIDs, diclofenac works by inhibiting the activity of the enzyme cyclooxygenase (COX) to disrupt prostaglandin synthesis [8]. However, alternative mechanisms of action of NSAIDs associated with the membrane activity of drugs are known [9,10,11]. For example, changes in lipid composition and/or membrane structure can lead to the development of various cardiovascular pathologies, including hypertension, myocardial infarction and thrombosis [12]. Diclofenac has demonstrated the ability to chemically interact with gastrointestinal protective-barrier phospholipids, which may contribute to its gastrointestinal toxicity [13,14]. Therefore, understanding the mechanisms of interaction of diclofenac with the plasma membrane at the molecular level can be extremely useful for understanding the therapeutic effect of this drug and developing ways to limit the side effects.

Among the huge variety of experimental approaches aimed at studying the molecular mechanisms of the interaction of NSAIDs with membranes [6,9,10,11,13,14,15,16,17,18,19,20,21,22], one can also use electron paramagnetic resonance (EPR) spectroscopy of spin-labeled drug molecules [23]. The spin-label EPR method provides different possibilities for studying molecules and intermolecular interactions [24]: rotational motions on the ns-timescale, molecular librations (sub-ns), polarity and H-bonding, accessibility and proximity, intermolecular and intramolecular separations (the nm-scale) and others. Also, the large magnetic moment of the electron spin makes it possible to reduce the studied concentrations to tenths of a mole percent, which can be close to therapeutic doses. 

This paper describes the synthesis of spin-labeled diclofenac and its interaction with a model membrane characterized by conventional and pulsed EPR. To introduce a nitroxide spin label into drugs that are derivatives of carboxylic acids, the use of transformations of carboxyl groups is the simplest way. Different variants of esterification and amidation reactions are well developed and widely used for the synthesis of esters and amides of many drugs [25,26,27,28]. For example, spin-labeled esters and amides of aspirin [29], ibuprofen [30], indomethacin [31] and diclofenac [32,33] were synthesized for their study using EPR. However, the loss of the carboxyl group in the original molecule of drugs is a significant drawback for their use as model objects of study. Indeed, the data from studies of the processes of action of NSAIDs [34], as well as the fact that the vast majority of them are derivatives of organic acids, convincingly indicate the exceptional importance of the carboxyl group.

Recently, we developed an alternative route to introduce the nitroxyl label into ibuprofen via the ethynylation of the benzene ring while retaining the carboxyl group and its associated molecular properties, particularly amphiphilicity [23]. Here, we report procedures for the synthesis of a novel diclofenac derivative in which the nitroxyl radical is linked to the benzene ring through an alkyne moiety distant from the carboxymethyl group.

## 2. Results and Discussion

### 2.1. Synthesis

**Scheme 1:**
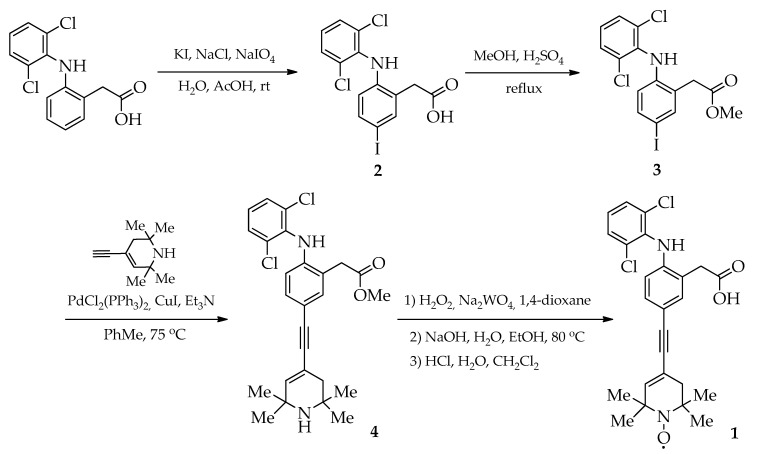
Synthesis of diclofenac-SL **1**.

The sequence of chemical transformation carried out in this work is shown in Scheme 1. To obtain spin-labeled diclofenac **1** (diclofenac-SL), we used a four-step synthesis, which is a new and simpler modification of our approach previously developed for ibuprofen [23]. Initially, iododiclofenac **2** was obtained in an 89% yield using the oxidative iodination of diclofenac with KI, NaIO_4_ and NaCl in acetic acid at room temperature. Then, iododiclofenac was converted into its methyl ester **3** in a 95% yield using an esterification reaction in boiling methanol with sulfuric acid. In the next step, a Pd/Cu-catalyzed Sonogashira coupling reaction of iodide **3** with 4-ethynyl-2,2,6,6-tetramethyl-1,2,3,6-tetrahydropyridine in the presence of Et_3_N at 75 °C produced precursor **4** (an 87% yield). Finally, the precursor was subjected to peroxidation in dioxane followed by saponification with aqueous sodium hydroxide to the target spin-labeled product **1** in a 34% yield. 

Diclofenac has a tendency for complex degradation when interacting with hydrogen peroxide in the presence of catalysts, such as peroxidases [35,36]. The poor yield of the key product in the last step indicates that the Na_2_WO_4_ acts similarly to peroxidases. In addition, there is a gradual reduction in the compound during purification and storage, which is typical of many nitroxide radicals. 

The structure and purity of synthesized compounds **1**–**4** were confirmed with ^1^H-NMR and ^13^C-NMR (see Supplementary Materials). According to the ^1^H-NMR spectra of the product, after a month of storage in the environment (see Figure S7), an accumulation of impurities in the region of 1.3 ppm (Me groups) was observed. 

### 2.2. EPR Spectra: Interaction with the POPC Membrane

**Figure 1:**
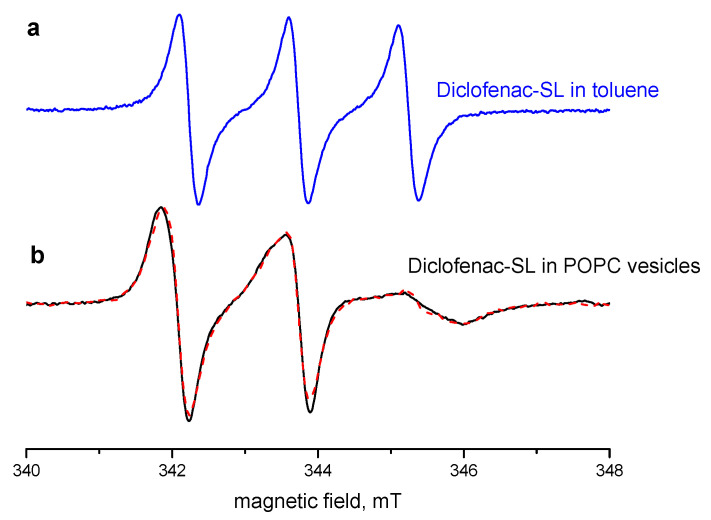
EPR spectra at room temperature of diclofenac-SL for its 5 mM solution in toluene (**a**) and in the presence of POPC vesicles at its concentration of 1 mol% (**b**). In the latter case, the samples were prepared either by mixing diclofenac-SL and POPC prior to the sample preparation (solid line), or by adding the solution of diclofenac-SL in DMSO to the prepared vesicles (dashed line).

The CW EPR spectra at room temperature (25 °C) obtained for diclofenac-SL are shown in Figure 1. In Figure 1a, the spectrum is given for diclofenac-SL dissolved in toluene at a concentration of 5 mM. This spectrum is characteristic of nitroxide in an isotropic medium in a state of fast tumbling motion of a spin label in a solution [24]. The value of the *g*-factor for this spectrum is 2.0023 ± 0.0001 and the hyperfine interaction constant is a = 1.5 ± 0.02 mT. 

Figure 1b shows the EPR spectra for diclofenac-SL in palmitoyl-2-oleoyl-sn-glycero-3-phosphocholine (POPC) multilamellar vesicles. Samples were prepared in two ways (see Section 3 below). In the first one, vesicles were prepared from the initial mixture of diclofenac-SL/POPC mixture (1:99 mol/mol). In the second, diclofenac dissolved in dimethyl sulfoxide (DMSO) was added to pure POPC vesicles. One can see from Figure 1b that the difference in the EPR spectra for the two different preparation methods is insignificant.

It is important that the spectra in Figure 1b show a significant decrease in the amplitude of the high-field component compared to the other two components (cf. Figure 1a). This decrease indicates a substantial retardation of the motion [24], which can take place only if the molecules are bound to the membrane. Thus, these data clearly indicate that diclofenac-SL is incorporated into the membrane.

### 2.3. Pulsed EPR: Location in the POPC Membrane

ESEEM spectroscopy is capable of providing information about the spatial position of the spin label [37,38,39,40]. The ESEEM effect is caused with an anisotropic hyperfine interaction of an unpaired electron of a spin label electron with neighboring nuclei. The ESEEM intensity depends on the distance *r* between the electron and nuclear spins as *r*^−6^, and disappears at distances greater than 1 nm [37,38,39,40]. The ESEEM frequencies are determined with the type of nucleus; therefore, if the membrane is hydrated with deuterium water, then ^2^H ESEEM allows you to distinguish the signal exclusively from water. Also, it depends on the number of the surrounding nuclei. Thus, ^2^H ESEEM makes it possible to determine the position of the spin label relative to the membrane surface: the larger the ESEEM signal, the closer the spin label is to the surface. 

The ^2^H ESEEM time traces *E*(*t*) for diclofenac-SL in D_2_O-hydrated POPC bilayers were refined from the background spin relaxation by dividing by the mean echo decay, <*E(t)*>, as described previously [37,38,39,40]:

(1) $$E_{n}(t)=\frac{E(t)}{<E(t)>}-1$$

**Figure 2:**
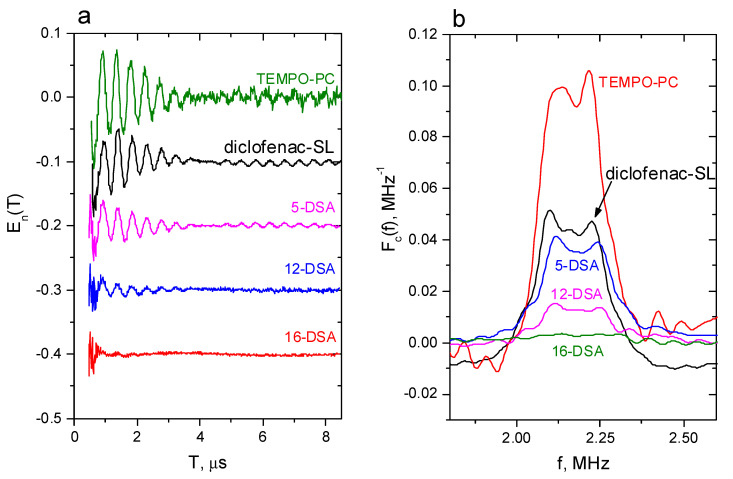
(**a**) ESEEM time traces for diclofenac-SL in D_2_O-hydrated POPC bilayer. For comparison, the analogous data for TEMPO-PC and 5 (12,16)-DSA (see text) are given. The data are vertically shifted for convenience. (**b**) Their Fourier transforms.

Figure 2a shows these refined ESEEM time traces for diclofenac-SL in D_2_O-hydrated POPC vesicles. Figure 2a also provides reference data on stearic acids, doxyl-spin-labeled (at the *n*-th carbon atom) positions along the carbon chain, *n*-DSA [23] and data [41] on 2-oleoyl-1-palmitoyl-sn-glycero-3-phospho(tempo)choline (TEMPO-PC). In TEMPO-PC, the spin label is attached to the polar lipid head. The corresponding Fourier transforms are shown in Figure 2b.

It can be seen that the TEMPO-PC sample shows the highest amplitude, which is obviously due to the fact that the spin label is located directly in the water shell. For spin-labeled stearic acids, *n*-DSA, the signal amplitude becomes smaller with an increasing *n*, which corresponds to immersion inside the membrane. For diclofenac-SL, the ESEEM amplitude shows a close proximity to that of the 5-DSA sample. So, we may conclude that the spin label is embedded into the membrane interior and that its mean position corresponds to the fifth position of the carbon atom in the lipid chain.

## 3. Materials and Methods

### 3.1. Chemical Analysis

^1^H and ^13^C spectra were recorded with a Bruker AV-500 (500 (^1^H) and 126 (^13^C) MHz) spectrometer in CDCl_3_ or DMSO-D_6_ solvents, by using residual signals of undeuterated solvents (CHCl_3_: δ = 7.26 ppm for ^1^H and δ = 77.16 for ^13^C ppm. DMSO: δ = 2.50 ppm for ^1^H) as an internal standard. Melting points were measured with an Electrothermal MEL-TEMP 1101D apparatus. HRMS was recorded with a Thermo Scientific DFS high-resolution mass spectrometer (Thermo Electron Corp., Waltham, MA, USA). The IR spectra were obtained on a Shimadzu IRTracer-100 instrument with a GS10802-X Quest ATR ZnSe Accessory (Specac, Kyoto, Japan). Column chromatography was carried out using silica gel (70−230 mesh ASTM). All reagents and solvents were obtained from commercial sources and used without special purification. Diclofenac was purchased from Sigma-Aldrich (China).

### 3.2. Synthesis and Characterization

4-Ethynyl-2,2,6,6-tetramethyl-1,2,3,6-tetrahydropyridine was obtained according to the method reported in [42].

*{2-[(2,6-Dichlorophenyl)amino]-5-iodophenyl}acetic acid* (**2**). KI (1.8 g, 10.8 mmol) was added in several portions to a stirred mixture of diclofenac (3.2 g, 10.8 mmol), NaCl (1.26 g, 21.5 mmol), NaIO_4_ (2.3 g, 10.8 mmol) and H_2_O (10 mL) in AcOH (60 mL) at room temperature for 24 h. After the disappearance of the iodine color, the precipitate was filtered, washed with H_2_O and dried in air. The crude product was purified with flash chromatography on silica gel (eluent: ethyl acetate). Yield 4.0 g (88%), white solid, mp 191–193 °C (toluene). ^1^H-NMR (500 MHz, DMSO-D_6_) δ: 7.53 (m, 3H), 7.35 (d, J = 8.4 Hz, 1H), 7.31 (s, 1H), 7.23 (m, 1H), 6.04 (d, J = 8.4 Hz, 1H), 3.68 (s, 2H) (see ^1^H-NMR in [43,44]). IR (film) cm^−1^: 3352 (NH), 3182, 3028, 2814 (OH), 1693 (C=O).
*Methyl {2-[(2,6-dichlorophenyl)amino]-5-iodophenyl}acetate* (**3**). A mixture of acid **2** (2.0 g, 4.75 mmol) and concentrated H_2_SO_4_ (1 mL) in methanol (45 mL) was stirred at reflux for 0.3 h. Then, the reaction mixture was cooled to room temperature; the precipitate was isolated using filtration, washed with methanol and dried in air. Yield 1.96 g (95%), white solid, mp 119–120 °C (methanol). ^1^H-NMR (500 MHz, CDCl_3_) δ: 7.53 (d, J = 1.5 Hz, 1H), 7.39 (dd, J = 8.5, 1.8 Hz, 1H), 7.35 (d, J = 8.0 Hz, 2H), 7.01 (t, J = 8.0 Hz, 1H), 6.94 (br.s, 1H), 6.28 (d, J = 8.5 Hz, 1H), 3.76 (s, 3H), 3.75 (s, 2H). ^13^C-NMR (126 MHz, CDCl_3_) δ: 172.24, 142.83, 139.44, 137.19, 136.95, 129.96, 129.08, 126.29, 124.78, 119.97, 84.13, 52.73, 38.16. IR (film) cm^−1^: 3321 (NH), 2949 (Me), 1714 (C=O). HRMS (ESI) *m*/*z*: [M]^+^ Calcd for C_15_H_12_Cl_2_INO_2_ 434.9284. Found 434.9285.
*Methyl {2-[(2,6-dichlorophenyl)amino]-5-[(2,2,6,6-tetramethyl-1,2,3,6-tetrahydropyridin-4-yl)ethynyl]phenyl}acetate* (**4**). Under an argon atmosphere, a mixture of iodide **3** (500 mg, 1.15 mmol), PdCl_2_(PPh_3_)_2_ (20 mg, 0.03 mmol) and CuI (10 mg, 0.05 mmol) in toluene (20 mL) was stirred and heated to 55 °C. Then, Et_3_N (8 mL) and 4-ethynyl-2,2,6,6-tetramethyl-1,2,3,6-tetrahydropyridine (270 mg, 1.65 mmol) were added, and the reaction mixture was stirred at 75 °C for 16 h. After cooling, toluene (100 mL) was added and the mixture was filtered. The extract was washed with 6 M of aqueous ammonia (2 × 200 mL) and dried over MgSO_4_. The crude product was purified with column chromatography on silica gel (eluent: toluene/ethyl acetate, 4:1). Yield 470 mg (87%), white solid, mp 150–151 °C (ethyl acetate). ^1^H-NMR (500 MHz, CDCl_3_) δ: 7.34 (m, 3H, H_Ar_), 7.19 (m, 1H), 7.11 (s, 1H), 7.01 (t, J = 8.0 Hz, 1H), 6.45 (d, J = 8.2 Hz, 1H), 6.05 (s, 1H), 3.76 (s, 2H), 3.75 (s, 3H), 2.87 (br.s, 1H), 2.07 (s, 2H), 1.25 (s, 6H), 1.20 (s, 6H). ^13^C-NMR (126 MHz, CDCl_3_) δ: 172.52, 142.71, 140.23, 137.06, 134.25, 131.34, 129.90, 129.04, 124.68, 123.59, 117.68, 116.46, 116.33, 89.61, 87.68, 52.70, 51.91, 49.71, 41.03, 38.46, 30.94, 29.75. IR (film) cm^−1^: 3348 (NH), 2961 (Me), 1745 (C=O). HRMS (ESI) *m*/*z*: [M]^+^ Calcd for C_26_H_28_Cl_2_N_2_O_2_ 470.1522. Found 470.1521.
*[4-{[3-(Carboxymethyl)-4-((2,6-dichlorophenyl)amino)phenyl]ethynyl}-2,2,6,6-tetramethyl-3,6-dihydropyridin-1(2H)-yl]oxidanyl* (**1**). A mixture of compound **4** (120 mg, 0.25 mmol), Na_2_WO_4_·2H_2_O (13 mg, 0.04 mmol), EDTA disodium salt (13 mg, 0.04 mmol) and 30% H_2_O_2_ (0.3 mL) in 1,4-dioxane (3 mL) was stirred at the ambient temperature for 14 days. The suspension was filtered, and the solvent was evaporated under a vacuum condition. Then, 2 M of NaOH (3 mL) was added, and the reaction mixture was stirred at 80 °C for 0.5 h. Next, the resulting mixture was diluted with ethanol (3 mL) and stirred at reflux for 0.25 h. After cooling, H_2_O (5 mL) and CH_2_Cl_2_ (15 mL) were added. At 0 °C, 1 M of HCl was added dropwise to the stirred mixture until the pH was neutral. The organic layer was separated and dried over MgSO_4_. The solvent was evaporated under a vacuum condition. The crude product was purified with column chromatography on silica gel (eluent: toluene/ethyl acetate, 5:1). Yield 40 mg (34%), white solid, mp 190–192 °C (toluene). ^1^H-NMR (500 MHz, CDCl_3_) δ: 7.51 (m, 4H), 7.10 (s, 1H), 6.95 (s, 1H), 6.46 (s, 1H), 3.98 (s, 2H). ^13^C-NMR (126 MHz, CDCl_3_) δ: 176.065, 140.80, 135.85, 130.19, 128.73, 128.02, 127.33, 123.77, 122.90, 119.32, 117.51, 38.66. IR (film) cm^−1^: 3300 (NH), 3042 (OH), 2928, 2851 (Me), 2203 (C≡C), 1695 (C=O). HRMS (ESI) *m*/*z*: [M]^+^ Calcd for C_25_H_25_Cl_2_N_2_O_3_ 471.1237. Found 471.1235.

### 3.3. Sample Preparations for EPR Investigation

For obtaining EPR spectra in the toluene solution, diclofenac-SL **1** was dissolved in toluene (Ekros-Analytica, St. Petersburg, Russia; distilled before use) at a concentration of 5 mM. Lipids’ POPC was from Avanti Polar Lipids (Birmingham, AL, USA). 

The vesicle samples were prepared in two ways. First, POPC and diclofenac-SL were dissolved separately in chloroform, then two solutions were mixed, so that the diclofenac-SL/POPC molar ratio was 1:99. The solvent was removed in the nitrogen stream, with the subsequent storing of the mixture under a vacuum for 4 h. Then, phosphate-buffered saline (pH 7.0) was added to the resulting powder in a proportion of 10:1. The sample was stirred, then stored for 2 h, and the resulting vesicles were centrifuged to remove the excess solvent. Instead of ordinary water, deuterium-substituted water was used in some measurements. 

For the second way of sample preparation, POPC vesicles were prepared in the same way but without diclofenac-SL, with the subsequent addition of a dimethylsulfoxide (DMSO) solution of diclofenac-SL (DMSO content was less than 10 vol% respective to the sample volume). The total diclofenac-SL mole content was 100 times smaller than the total lipid mole content. 

The samples were placed in glass EPR tubes with an outer diameter of 3 mm and examined either at room temperature (25 °C) or at 80 K. In the latter case, the samples were quickly frozen using immersion in liquid nitrogen.

### 3.4. EPR Measurements

Conventional EPR spectra were obtained at room temperature with a Bruker ESP 380E spectrometer operating at a modulation amplitude of 0.01 mT, with the output microwave (MW) power of 100 mW, the MW attenuation set up to 25 dB and the sweep and constant times of 60 s and 46 ms, respectively. A Bruker ER 4118 X-MD-5 dielectric resonator was used. In pulsed EPR studies, an X-band Bruker ELEXSYS E580 EPR spectrometer was used equipped with a split-ring Bruker ER 4118 X-MS-3 resonator and an Oxford Instruments CF-935 cryostat. 

The three-pulse ESEEM sequence (π/2)-τ-(π/2)-t-(π/2)-τ-echo was employed, with excitation at the maximum of the echo-detected EPR spectrum. The pulse lengths were 16 ns and the time delay τ was 204 ns, and the time delay t was scanned from 300 ns to 10 μs, with a 12 or 16 ns time step. The resonator was cooled with a stream of cold nitrogen gas. The temperature was controlled with a nitrogen flow stabilized by a Bruker ER4131VT temperature controller. The sample temperature was kept near 80 K.

## 4. Conclusions

This work shows that the NSAID diclofenac can be spin-labeled through a simple four-step sequence that includes iodination, esterification, Sonogashira cross-coupling, oxidation and saponification. The obtained CW EPR data indicate that diclofenac-SL binds to model lipid membranes. The ESEEM spectroscopy shows that diclofenac molecules are located under the polar heads of lipids with an average position near the fifth position of the carbon of the lipid chain.

Note that a standard problem with the labeling approach is that the label distorts the original molecule. In our case, the main structural properties of the diclofenac molecule are a polar carboxyl residue and a Cl-containing aromatic ring, and these properties are not violated in the suggested labeling scheme. 

On the other hand, the advantages of spin-label EPR are that it makes it possible to reduce the studied concentrations to close to therapeutic doses and that it provides information of a different kind at the molecular level, such as molecular motions; polarity and H-bonding; accessibility and proximity; and nanoscale structure. With regard to the latter possibility, it has been recently shown that pulsed EPR in the version of double electron–electron resonance (DEER, also known as PELDOR) allows for obtaining data on the nanoscale clustering of mono-spin-labeled molecules [45,46,47,48]. 

## Data Availability

Data are contained within the article.

## Supplementary Materials

The following supporting information can be downloaded at: https://www.mdpi.com/article/10.3390/molecules28165991/s1, Figure S1: ^1^H-NMR spectrum of compound **2** in DMSO-D_6_; Figures S2–S5: ^1^H-NMR spectrum of compounds **3**–**4** in CDCl_3_; Figure S6: ^1^H-NMR spectrum of compound **1** in CDCl_3_; Figure S7: ^1^H-NMR spectrum of compound **1** in CDCl_3_ after 1 month of storage in the environment; Figure S8: ^13^C-NMR spectrum of compound **1** in CDCl_3_.
