# Supplementary material for: Spin-Labeled Diclofenac: Synthesis and Interaction with Lipid Membranes
Source: Molecules. 2023 Aug 10;28(16):5991. doi: 10.3390/molecules28165991 (PMC10458756; doi:10.3390/molecules28165991)
Supplement: Supplementary file 1 [file molecules-28-05991-s001.zip › molecules-2544103-supplementary.pdf]

Supplementary Materials

Spin-labeled Diclofenac: Synthesis and Interaction with Lipid Membranes

Denis S. Baranov,[1] Anna S. Kashnik,[1] Anastasiya N. Atnyukova,[2] Sergei A. Dzuba[1*]

[1] Voevodsky Institute of Chemical Kinetics and Combustion, SB RAS, 630090 Novosibirsk, Russian Federation;
[2] Department of Physics, Novosibirsk State University, 630090 Novosibirsk, Russian Federation.
* Correspondence: dzuba@kinetics.nsc.ru)

Table of Contents

Figure S1. ^{1}H-NMR spectrum of compound **2** in DMSO-D_6.......................... S2

Figure S2. ^{1}H-NMR spectrum of compound **3** in $CDCl_3$.............................. S3

Figure S3. ^{13}C-NMR spectrum of compound **3** in $CDCl_3$............................ S3

Figure S4. ^{1}H-NMR spectrum of compound **4** in $CDCl_3$.............................. S4

Figure S5. ^{13}C-NMR spectrum of compound **4** in $CDCl_3$............................ S4

Figure S6. ^{1}H-NMR spectrum of compound **1** in $CDCl_3$.............................. S5

Figure S7. ^{1}H-NMR spectrum of compound **1** in $CDCl_3$ after one month of storage in the environment.. S5

Figure S8. ^{13}C-NMR spectrum of compound **1** in $CDCl_3$.............................. S6

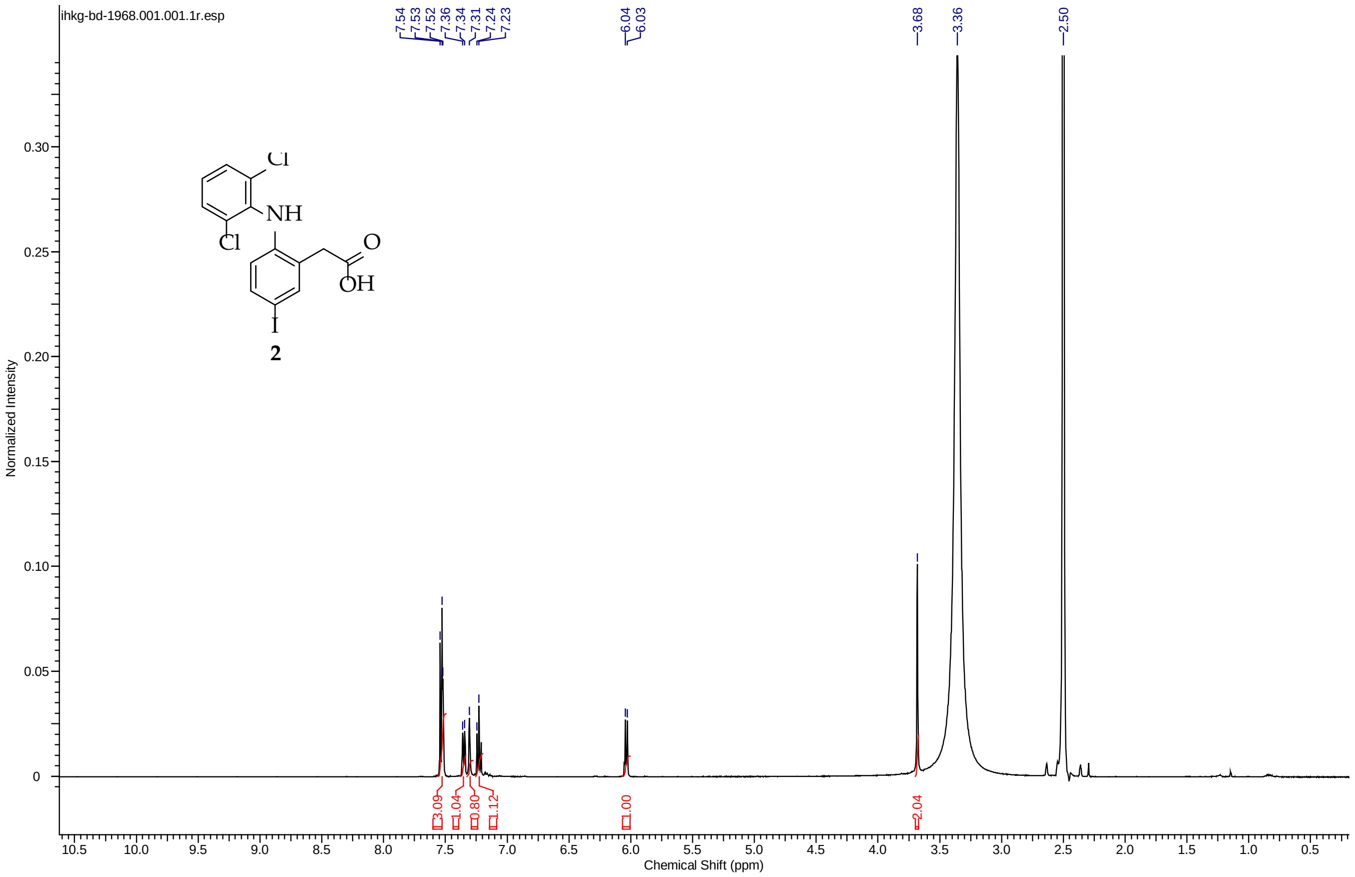

Figure S1. ^{1}H-NMR spectrum of compound **2** in DMSO-D$_6$.

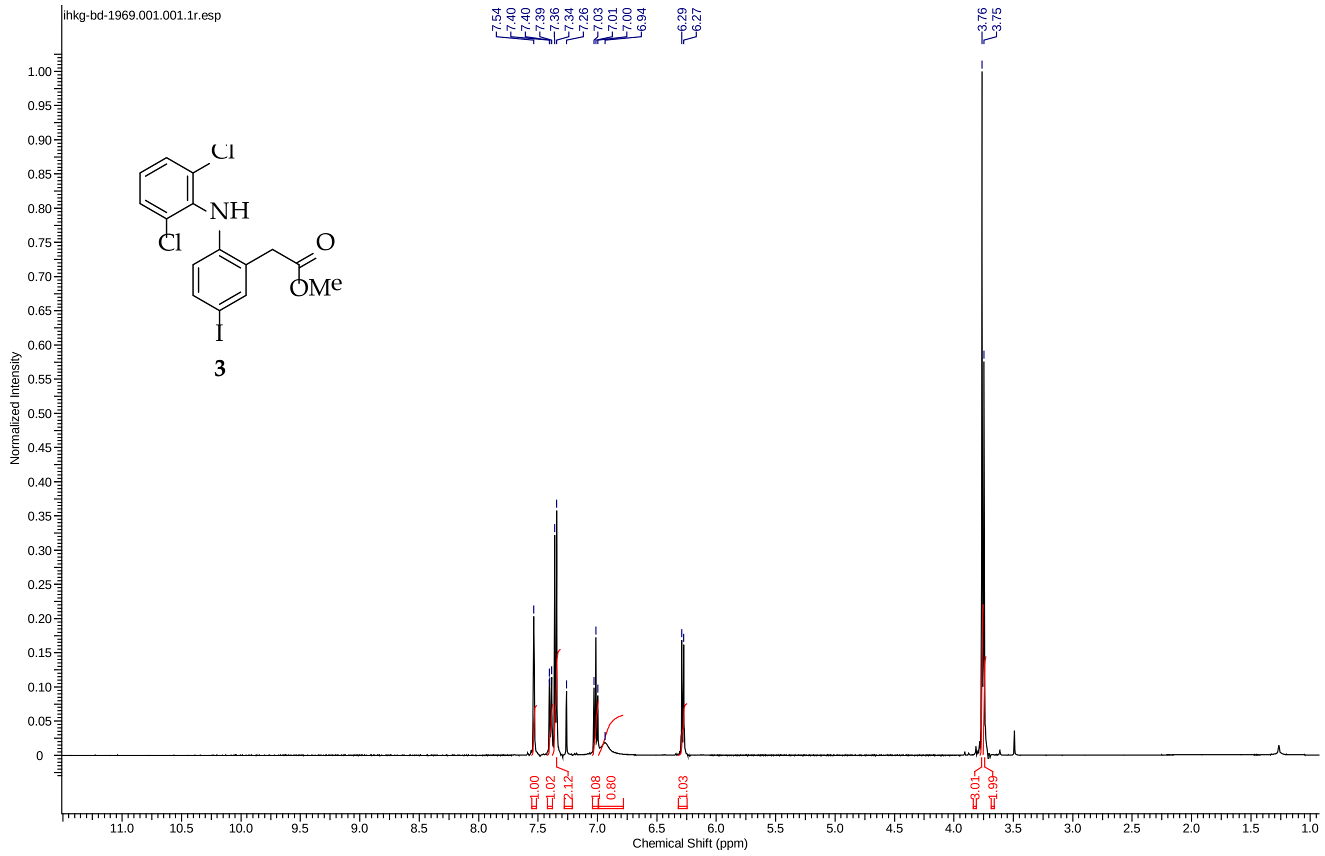

Figure S2. ^{1}H-NMR spectrum of compound **3** in $CDCl_3$.

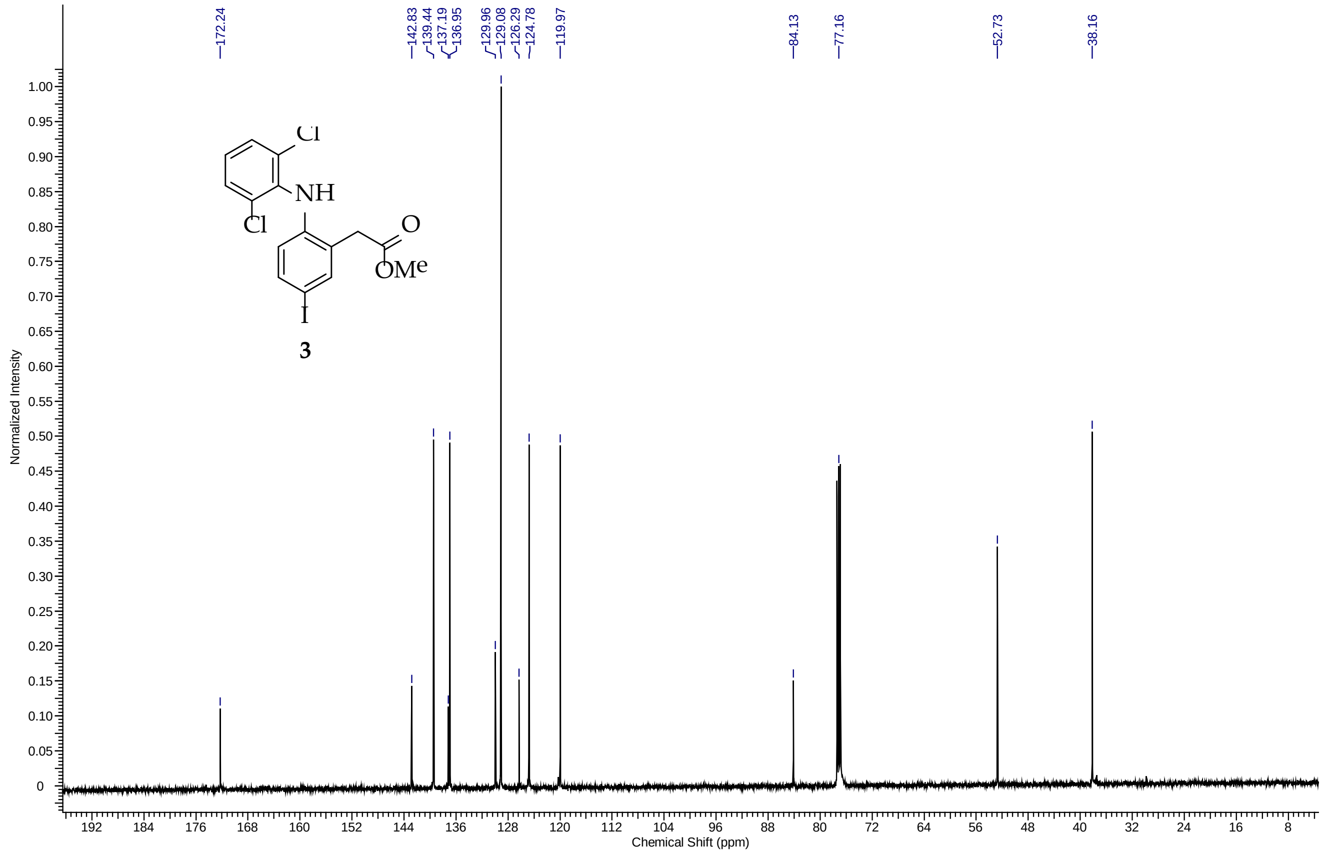

Figure S3. ^{13}C-NMR spectrum of compound **3** in $CDCl_3$.

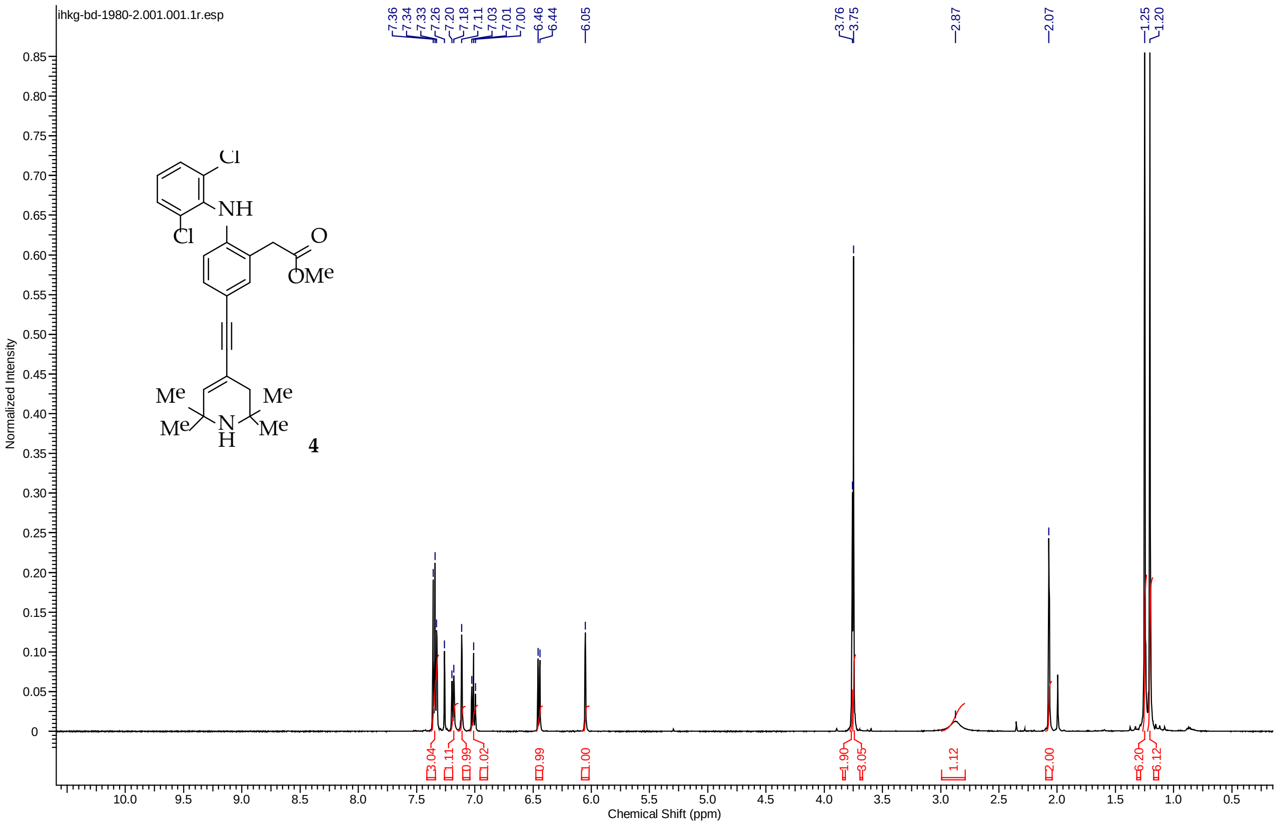

Figure S4. ^{1}H-NMR spectrum of compound **4** in $CDCl_3$.

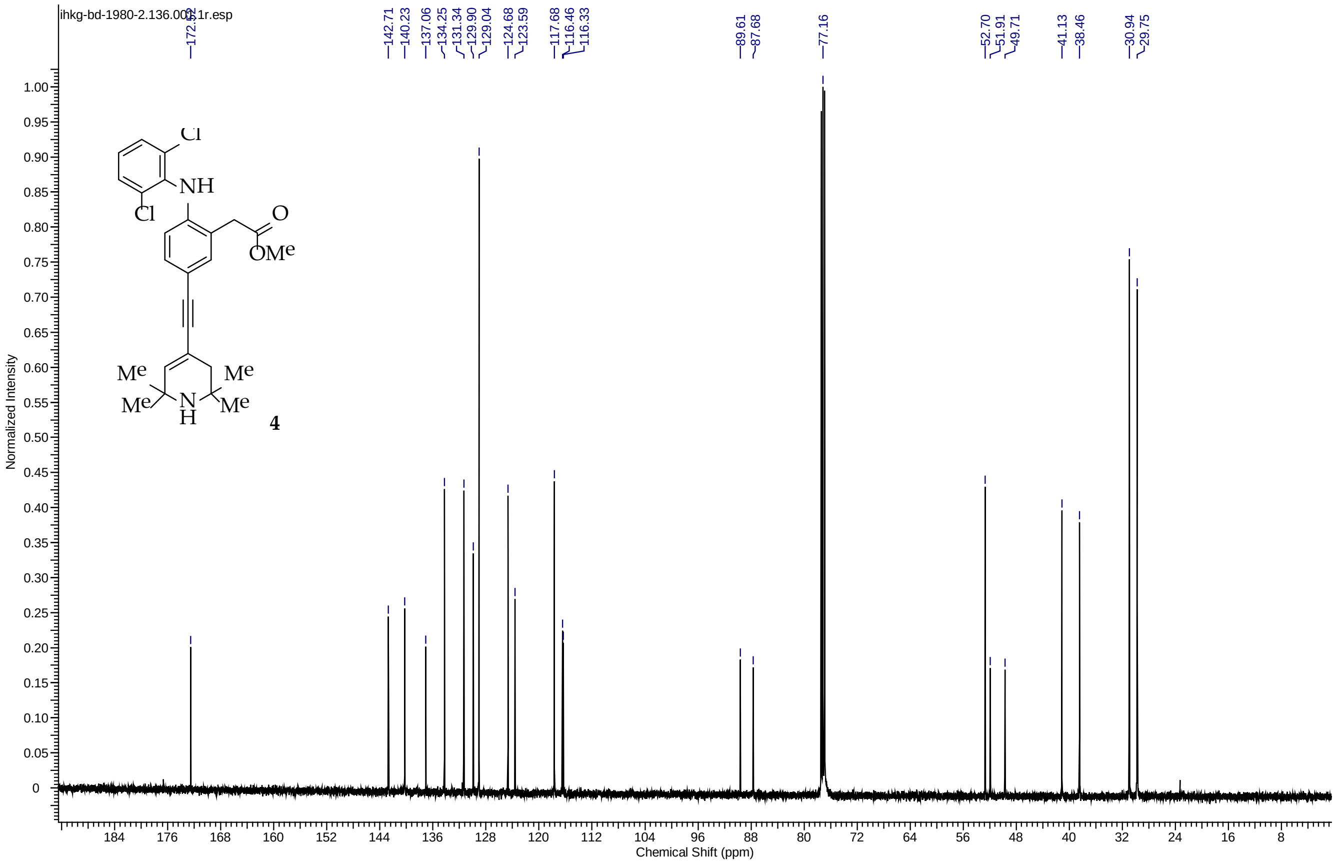

Figure S5. ^{13}C-NMR spectrum of compound **4** in $CDCl_3$.

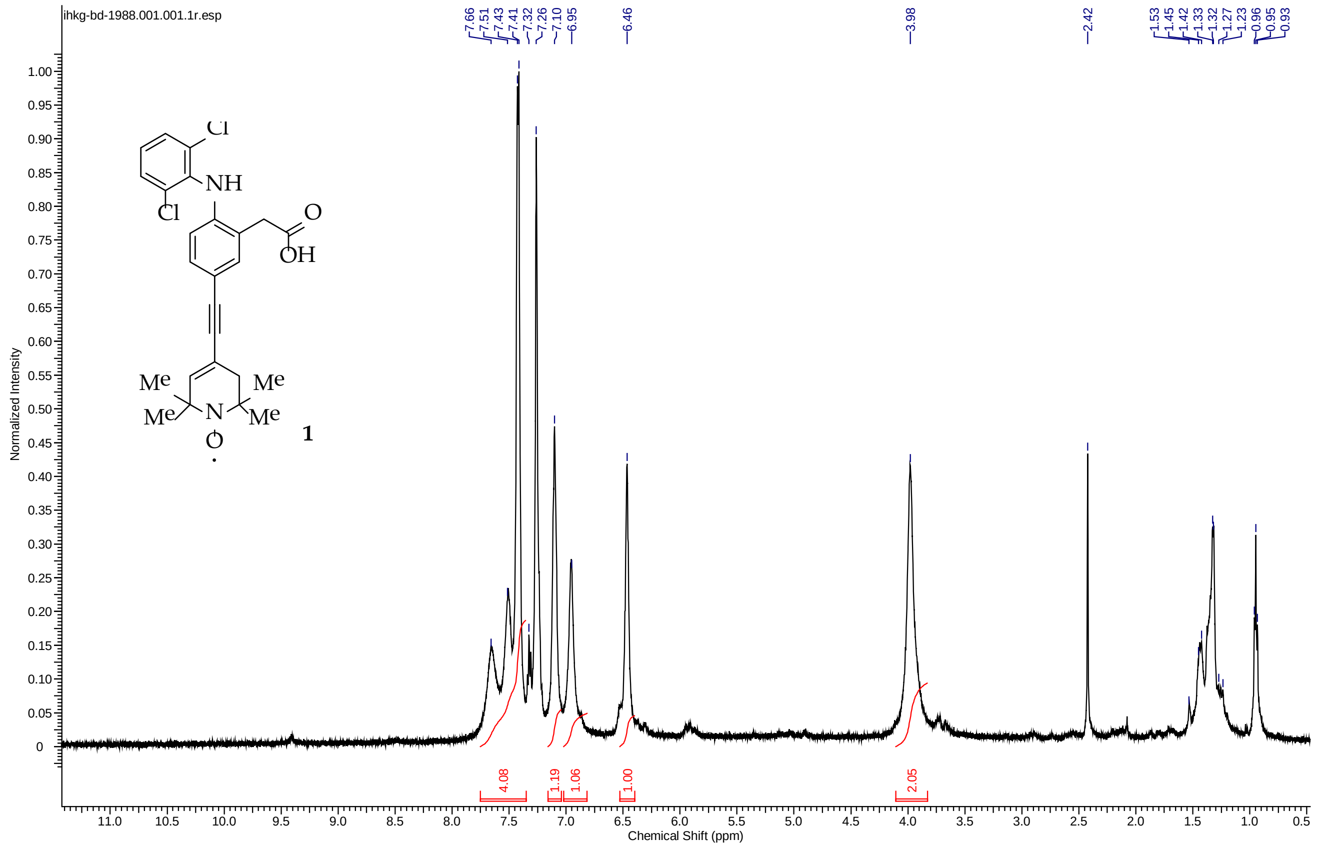

Figure S6. ^{1}H-NMR spectrum of compound **1** in $CDCl_3$.

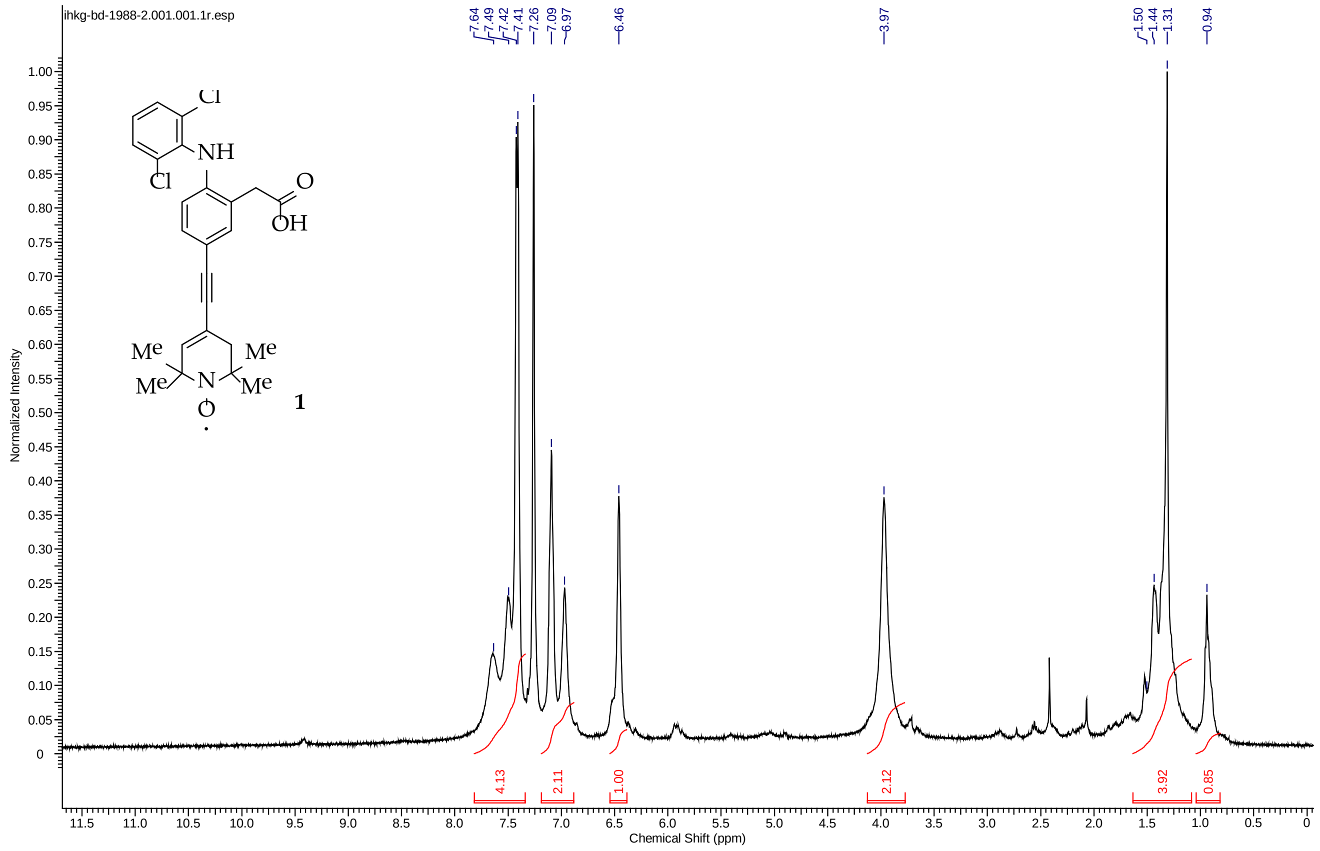

Figure S7. ^{1}H-NMR spectrum of compound **1** in $CDCl_3$ after one month of storage in the environment.

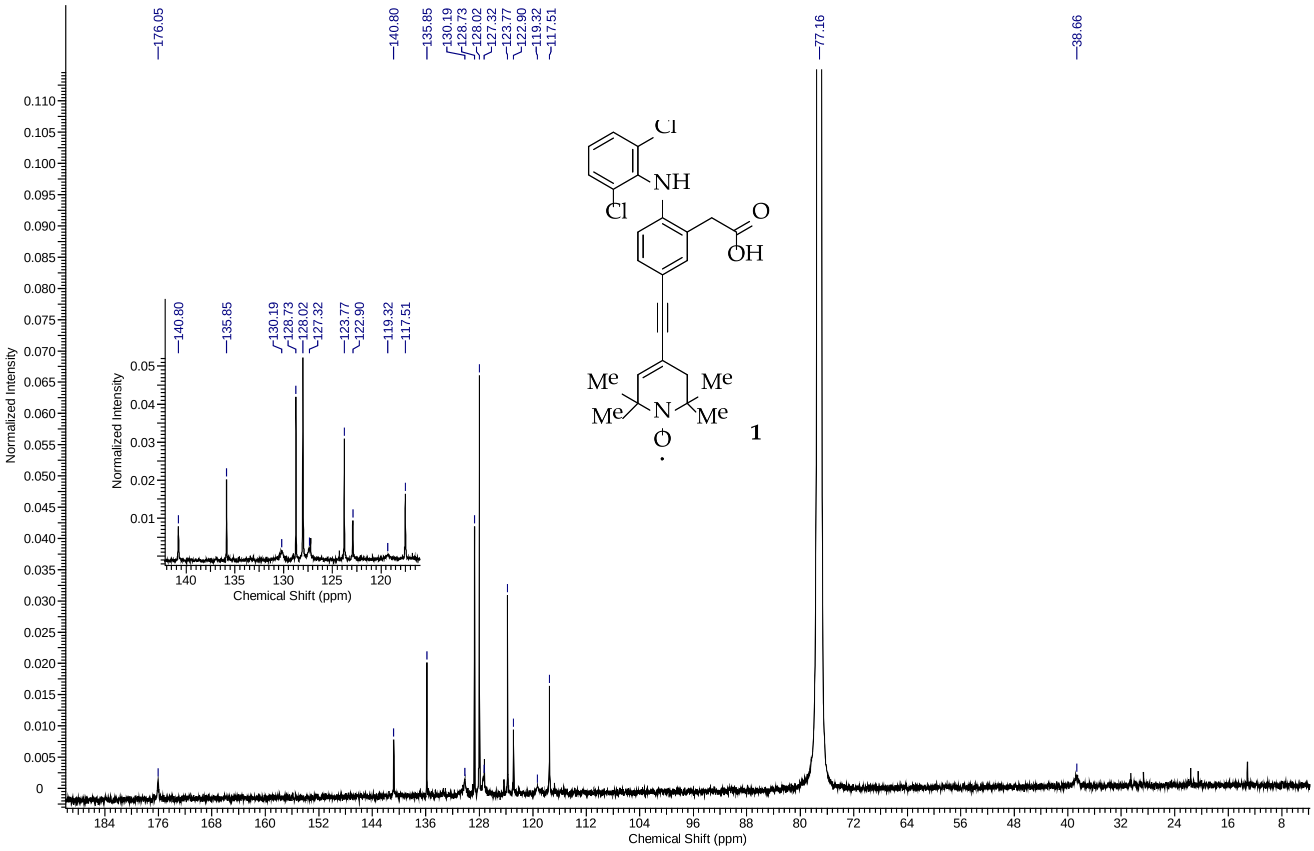

Figure S8. ^{13}C-NMR spectrum of compound **1** in $CDCl_3$.
